# Factors Associated With Poor Long-Term Outcomes After Emergency Department Intubation

**DOI:** 10.7759/cureus.15178

**Published:** 2021-05-22

**Authors:** Caroline A Colleran, Craig T Brewster, Andrew J Kroemer, Brendan Miccio, Calvin A Brown III, Jestin N Carlson

**Affiliations:** 1 Emergency Medicine, Allegheny Health Network (AHN) Saint Vincent Hospital, Erie, USA; 2 Emergency Medicine, Lake Erie College of Osteopathic Medicine, Erie, USA; 3 Emergency Medicine, Brigham and Women's Hospital, Harvard Medical School, Boston, USA

**Keywords:** endotracheal intubation, survival, hospital discharge, intubation, critical care, rsi

## Abstract

Introduction

While immediate complications of ED patients undergoing endotracheal intubation (ETI) have been explored, the relationship between ED ETI and patient status at hospital discharge is unknown.

Methods

We performed a retrospective review of all intubations performed in our ED for one calendar year in adult patients (>18 years of age). We abstracted patient and ETI factors (indication, complications, etc.) to determine their impact on patient outcomes. We defined a poor outcome as either (1) death or discharge to a nursing home if admitted to the hospital from home or (2) death if admitted to the hospital from a nursing home. We examined the univariate odds ratios for poor outcomes.

Results

We identified 122 intubations; 64 (52.5%) had a poor outcome and 58 (47.5%) did not have a poor outcome. Age in years (odds ratio [OR] 1.04, 95% confidence interval [CI] 1.02-1.07) and ETI performed for an indication of "cardiac arrest" (OR 4.49, 95% CI 1.55-13.01) were the only variables associated with a poor outcome. Other patients and intubation variables were not associated with a poor outcome including; gender, difficult airway characteristics, intubator skill level, first attempt success, airway complications, and post-intubation hypoxia or hypotension.

Conclusion

In our sample from a single ED, over 50% of patients who undergo ED ETI either died in the hospital or failed to return home. While age and an ETI indication of "cardiac arrest" were associated with poor outcomes, future work is required to validate our findings in a larger cohort.

## Introduction

Endotracheal intubation (ETI) and mechanical ventilation are performed in acutely ill and injured patients for a variety of different conditions and the frequency of mechanical ventilation has been increasing [1]. ETI and invasive mechanical ventilation is a high-risk procedure that can adversely affect patients both through the short-term risk of ETI (e.g., hypotension and peri-intubation cardiac arrest, etc.) and the longer-term complications of mechanical ventilation such as acute lung injury [2-5]. In addition to these complications, the mortality of patients on mechanical ventilation is significant, exceeding 35% in non-surgical patients [4]. In addition, patients who do survive hospital discharge after mechanical ventilation can suffer from increased morbidity and decreased ability to complete activities of daily living [6].

With more adults undergoing invasive mechanical ventilation in ED and the potentially negative outcomes for a significant portion of these patients, it is important to understand the factors that adversely affect intubated patients and may aid in management decisions. Patients intubated in the emergency department are often intubated due to acute respiratory failure from pneumonia, chronic obstructive pulmonary disease (COPD), or heart failure [7]. These patients can have a variety of other co-morbid conditions affecting their hospital stay with only 24% of patients who had invasive mechanical ventilation being discharged straight to home instead of a nursing or rehabilitation facility [7]. While these data provide insight to potential predictors of poor patient outcomes, components of the ED ETI were not able to be examined in this administrative dataset. ED ETI performance impacts short-term outcomes with up to 4% of ED ETIs suffering from cardiac arrest [2]. It is unknown that how ED ETI performance impacts longer-term patient outcomes. A better understanding of ED ETI performance and how it impacts outcomes could guide targeted interventions to help improve specific aspects of ED ETI (e.g., improve first attempt success). This may also help providers better identify patients, intubated in the ED, who may suffer poor outcomes, allowing for deeper discussions regarding goals of care for resuscitative efforts and in-hospital care. Prior studies have examined trends and outcomes in those mechanically ventilated but have not examined ETI risk factors associated with poor patient outcomes.

Given the increasing frequency of ETI, the high mortality rate of mechanical ventilation, and the potential long-term effects of intubation, there is a need to further examine what risk factors can adversely affect patient outcomes. We sought to determine factors associated with poor patient outcomes after ED intubation.

## Materials and methods

A retrospective chart review was performed utilizing a prospectively collected database examining patient outcomes in adults undergoing endotracheal intubation in a single emergency department.

Study design and setting

We conducted a retrospective chart review of adult patients (age >18 years) undergoing endotracheal intubation in a single urban, community teaching emergency department with an annual ED volume of ~60,000 visits. This ED is a participating center in the National Emergency Airway Registry (NEAR) database and prospectively tracks all ETIs in our ED [8]. The details of NEAR have been described previously [8]. Briefly, NEAR prospectively collects information on patient demographics (age, gender, weight, and body habitus) and ETI characteristics (indication for ETI; number of attempts; technique used; medications used during ETI; complications during ETI [hypoxia, hypotension, aspiration, arrest]; and complication after ETI [hypotension or hypoxia]). Additional patient demographics (initial place of residence) and disposition (died, home, rehab/nursing facility) were abstracted from the electronic health record as it is not recorded in NEAR. This work was approved by our institutional review board.

Patient selection

We included all encounters with intubations in the ED for adult patients from May 1, 2018, to April 30, 2019. This start date was selected as we implemented a new electronic medical record in March of 2018. We selected a start date after that point (May 1, 2018) to ensure consistency with data abstract from a single electronic medical record. We excluded encounters where the patient was intubated outside of the hospital (i.e., via emergency medical services), transferred to another facility where we were unable to determine the final disposition or where the patient was <18 years of age.

Outcomes

Our primary outcome was a poor outcome defined as (1) death or discharge to a nursing home if admitted to the hospital from home or (2) death if admitted to the hospital from a nursing home.

Data abstraction

Retrospective data were abstracted via two trained research assistants (BM and AK) who met with the investigative team (CB and JNC) regularly to ensure appropriate chart abstraction. All data were collected utilizing a standard data collection template. In order to ensure consistent data abstraction, we calculated the inter-rater agreement using Cohen’s kappa by having each research assistant evaluate 10% of the other research assistant’s charts. Each research assistant was blinded to the data abstracting done by the other research assistant.

Power and sample size

Given our ED volume of ~60,000 visits annually and national rates of ETI estimates of 2.7/1000 ED visits, we anticipated roughly 160 intubations during the proposed time period [9]. Assuming 10% of ETIs were performed in patients <18 years of age and in patients transferred with the unknown final disposition, we anticipated roughly 145 ETIs available for analysis. With estimated inpatient mortality of 35%, we anticipated a minimum of 50 patients with a poor outcome [4].

Statistical analysis

We report demographic data using descriptive statistics (medians with interquartile range [IQR] and means with standard deviations [SD]). We reported the univariate association between candidate predictor variables and our primary outcome (worsened disposition) using logistic regression. Stata version 12.1 (College Station, TX: StataCorp. LLC) was used for all analyses. We report the agreement between reviewers using Cohen’s kappa for our primary outcome of poor outcome and agreement was 100% between the two reviewers. 

## Results

**Figure 1:**
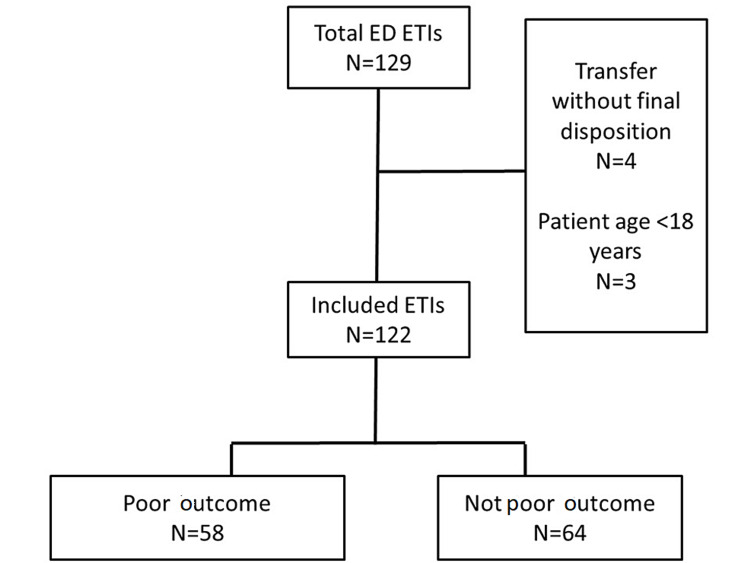
Study Flow Diagram of Included Patients ED - emergency department; ETI - endotracheal intubation

We identified 122 intubations between May 1, 2018, and April 30, 2019. Of those, 64 (52.5%) had a "poor outcome" and 58 (47.5%) did not have a poor outcome (Figure 1). The majority of ETIs in both groups were performed in women (poor outcome 40/64; 62.5%; not a poor outcome 38/58; 65.5%). The most common indication for patients with a poor outcome was "cardiac arrest" (19/63; 30.2%) while "non-overdose mental status change" and "overdose" were the most common indications for patients without a poor outcome (11/53; 19.3% for each) (Table 1). In many instances, the provider anticipated a difficult airway (poor outcome 31/64; 48.4%; not a poor outcome 26/58; 44.8%).

**Table 1:** Intubation Characteristics SD - standard deviation; kg - kilograms; COPD - chronic obstructive pulmonary disease; RSI - rapid sequence intubation; ED - emergency department; PGY - post-graduate year Totals may not equal 100% due to rounding. *For clarity, only the top five most frequent indications are shown.

	Patients Who Did Not Have a Poor Outcome N = 58 (47.5%)	Patients With a Poor Outcome N = 64 (52.5%)
Mean Age in Years (SD)	55 (18.4)	67.9 (15.7)
Female Gender	38 (65.5%)	40 (62.5%)
Estimated Weight in kg (SD)	87.2 (27.6)	86 (24)
Medical Indications for Intubation* (N= 120)	N = 57 (47.5%)	N = 63 (52.5%)
Cardiac Arrest	5 (8.8%)	19 (30.2%)
COPD	8 (14.0%)	9 (14.3%
Non-Overdose Mental Status Change	11 (19.3%)	6 (9.5%)
Overdose	11 (19.3%)	5 (7.9%)
Shock (Sepsis)	4 (7.0%)	5 (7.9%)
Initial Airway Difficulty	26 (44.8%)	31 (48.4%)
Neck Immobility	6 (10.3%)	12 (18.8%)
Mallampati (N= 121)	N= 58 (47.5%)	N= 63 (51.6%)
Class 1	8 (13.8%)	3 (4.8%)
Class 2	9 (15.5%)	11 (17.4%)
Class 3	11 (19.0%)	10 (15.9%)
Class 4	5 (8.6%)	6 (9.5%)
Not Assessed	25 (43.1%)	33 (52.4%)
Mouth Opening (N=121)	N= 58 (47.5%)	N= 63 (51.6%)
Normal (3+ Fingers)	19 (32.8%)	17 (27.0%)
Reduced	13 (22.4%)	17 (27.0%)
Not Assessed	26 (44.8%)	29 (46.0%)
Thyromental Distance (N=121)	N= 58 (47.5%)	N= 63 (51.6%)
One Finger	1 (1.7%)	2 (3.2%)
Two Fingers	9 (15.5%)	13 (20.6%)
Three Fingers	10 (17.2%)	5 (7.9%)
Not Assessed	38 (65.5%)	43 (68.3%)
Obstruction Present	6 (10.3%)	4 (6.3%)
Facial Trauma	0 (0.0%)	1 (1.6%)
Blood In Airway	6 (10.3%)	15 (23.8%)
RSI Paralytic (N= 99)	N= 55	N= 44
Rocuronium	53 (96.4%)	43 (97.7%)
Succinylcholine	1 (1.8%)	0 (0.0%)
Vecuronium	1 (1.8%)	1 (2.3%)
Intubator Level		
PGY-1	25 (43.1%)	25 (39.1%)
PGY-2	19 (32.8%)	18 (28.1%)
PGY-3	6 (10.3%)	11 (13.9%)
PGY-4	7 (12.1%)	8 (12.3%)
Attending	1 (1.7%)	2 (2.5%)
First Attempt Success	52 (89.7%)	54 (84.4%)
Adverse Events	N= 58	N= 64
Hypoxia	4 (6.9%)	3 (4.7%)
Post Intubation Hypotension	2 (3.4%)	2 (3.1%)
Vomiting	0 (0.0%)	1 (1.6%)
Bradycardia	0 (0.0%)	1 (1.6%)
Cardiac Arrest	0 (0.0%)	3 (4.7%)
Oxygen Desaturation	5 (8.6%)	6 (9.4%)

In the univariate analysis, age in years was associated with an increased odds of a poor outcome (odds ratio [OR] 1.04, 95% confidence interval [CI] 1.02-1.07). Other patient variables were not associated with a poor outcome including gender (OR 1.14, 95% CI 0.54-2.39) and estimated weight in kilograms (OR 1, 95% CI 0.98-1.01). Given the limited incidence of difficult airway characteristics and adverse events, these were each collapsed into a single variable. In addition, we report the three most common indications for intubation for the univariate analysis and (Table 2). ETI performed for the indication of "cardiac arrest" was associated with a poor outcome (OR 4.49, 95% CI 1.55-13.01). None of the other intubation characteristics were associated with increased odds of poor outcome; any difficult airway characteristics (OR 1.15, 95% CI 0.57-2.36), intubator level (OR 1.13, 95% CI 0.84-1.53), first attempt success (OR 0.62, 95% CI 0.21-1.83), any airway complications (OR 1.74, 95% CI 0.76-3.98), post-intubation hypoxia (OR 1.43, 95% CI 0.41-5.04), and post-intubation hypotension (OR 2.38, 95% CI 0.87-6.54).

**Table 2:** Odds Ratio for Patients With Poor Outcomes CI - confidence interval

	Odds Ratio (95% CI)
Age (Years)	1.04 (1.02-1.07)
Gender	1.14 (0.54-2.39)
Indications for Intubation	
Cardiac Arrest	4.49 (1.55-13.01)
Non-Overdose Mental Status Change	0.44 (0.15-1.28)
Overdose	0.36 (0.17-1.11)
Any Difficult Airway Characteristics	1.15 (0.57-2.36)
Intubator Skill Level	1.13 (0.84-1.53)
First Attempt Success	0.62 (0.21-1.83)
Any Airway Complication	1.74 (0.76-3.98)
Post-Intubation Hypoxia	1.43 (0.41-5.04)
Post-Intubation Hypotension	2.38 (0.87-6.54)

## Discussion

The decision of how to manage the airway of a patient in the emergency department can be challenging. Given the increasing frequency of mechanical ventilation, it is necessary to better understand the potential impact of the decision to pursue ETI and mechanical ventilation in these circumstances and assist with articulating the prognosis and risks to patients, families, and other healthcare proxies. In our study, 53% of patients had a poor outcome after intubation. We believed complications associated with airway management may have been associated with poor outcomes (e.g., post-intubation hypotension); however, only age and an indication of "cardiac arrest" were associated with a poor outcome in our evaluated factors.

Previous studies looking at intubation mortality in the hospital have focused on the epidemiology of mechanical ventilation, specifically on older adults and on specific diseases requiring intubation [7,10]. Our study looked at data for all adult patients requiring intubation and examined specific risk factors surrounding ED ETI that could be associated with increased mortality after intubation. Our findings suggest that increased age and the indication for ETI are the primary factors associated with an increased risk of mortality after intubation and confirm the results of prior studies showing in-hospital mortality is higher in older adults [3].

We did not identify an association between other ETI factors and poor outcomes, including intubator level, airway complications, first attempt success, difficult airway characteristics, and post-intubation hypotension or hypoxia. While we believe this is primarily due to our sample size, our results do not dispute the importance of these variables when managing the airway of critically ill patients especially given the importance of various ETI characteristics such as first attempt success as highlighted in other work [11]. These factors may have an impact on the subsequent care, immediate issues, or complications that may arise both in the ED and in the intensive care unit. These factors are still important in airway management and may impact other outcomes not examined here (e.g., rates of ventilator-associated pneumonia, number of days of mechanical ventilation, etc.). We believe our results may help to inform future, larger studies looking at the impact of ED ETI on patient outcomes.

Other studies examining the long-term outcome of patients admitted for critical illness have found that, unless a patient had a normal functional status prior to hospital admission, roughly 40% have a severe functional disability at hospital discharge [12]. Our results suggest this number may be even higher in patients undergoing ED ETI as we identified over half of patients in our study had poor outcomes. Our results do not suggest that ETI worsens outcomes and instead, is likely a marker of illness severity. As we identified that age was associated with an increased odds of a poor outcome, it is important to consider patient preferences in older populations. For example, in seriously ill adults greater than 60 years old, roughly 74% report they would not want treatment if the predicted survival included severe functional impairment [13]. Given the significant number of patients identified in our study who had a poor outcome, defined as either died or went to a nursing home instead of back to their homes, we believe that this information may help to inform ED staff of this outcome that can then be used during the course of a discussion with patients, families, and other medical providers as to possible poor outcomes associated with ED intubation. 

Limitations

Our study has several limitations. We utilized data from only one ED that could affect its generalizability. Future studies, utilizing a large sample will be needed to sharpen these findings. Our dataset only provides information on discharge location. We did not include other important post-hospitalization outcomes such as functional status and readmission. Future prospective studies should be completed to evaluate functional status at discharge and other long-term outcomes such as functional status at 30-days after ED ETI and 1-year outcomes. We did not assess patients’ functional status prior to arrival although we were able to abstract from the ED note if the patient presented from a nursing home, rehabilitation facility, or home as this is regularly reported in our ED documentation and were able to identify this in all 122 cases. We did not have a structured functional status at discharge such as modified Rankin Scale or Cerebral Performance Category. Future, prospective studies should examine these functional outcomes at hospital discharge. Given the heterogeneity in the indications for ETI, we did not evaluate the severity of illness or other ED-based treatment variables (e.g., time to antibiotics for patients with sepsis) that could affect mortality for specific critical illnesses. We did not examine patients who were initially placed on non-invasive positive pressure ventilation (e.g., bilevel positive airway pressure [BiPAP]) and then ultimately intubated in another setting such as the intensive care unit. While we attempted to limit the variability in chart abstraction and had a Cohen’s kappa of 1.00 for our outcome of interest on review of the electronic health record, variables of the NEAR data collection were inputted by the person performing the intubation and we are unable to provide additional details regarding the intubation attempt. We did not perform a multivariable model. Multivariable models require an understanding of valid key factors to be inputted into the model [14]. As there are limited data examining the impact of ED ETI on patient disposition, we performed a univariate analysis and future work will be needed to better inform the development of complex multivariable models. While we evaluated post-intubation vital signs, we did not have exact vital signs prior to the intubation as the timing of the intubation is unclear from our electronic medical record. Finally, we did not have enough patients with individual difficult airway characteristics to evaluate each characteristic independently. 

## Conclusions

In our sample from a single ED, over 50% of patients who undergo ED ETI have a poor outcome with age and indication for ETI ("cardiac arrest") associated with a poor outcome. While other ETI factors were not associated with a poor outcome, future work is required to validate our findings in a larger cohort.
